# Management of Humeral Defects in Anterior Shoulder Instability

**DOI:** 10.2174/1874325001711011011

**Published:** 2017-08-31

**Authors:** Maria Valencia Mora, Miguel Ángel Ruiz-Ibán, Jorge Diaz Heredia, Raquel Ruiz Diaz, Ricardo Cuéllar

**Affiliations:** 1 Hospital Fundación Jiménez Diáz, Madrid, Spain; 2 Hospital Universitario Ramón y Cajal; Madrid, Spain; 3 Hospital Universitario San Sebastían, San Sebastían, Spain

**Keywords:** Shoulder, Instability, Humeral defect, Remplissage, Autograft, Osteotomy

## Abstract

**Background:**

A Hill Sachs lesion is a posterior-superior bony defect of the humeral head caused by a compression of the hard glenoid rim against the soft cancellous bone in the context of an anterior instability episode. The presence of these humeral defects increases with the number of dislocations and larger lesions are associated with a greater chance of development of recurrent instability and recurrence after surgery. Also its location and pattern, in particular the so-called engaging Hill-Sachs, are associated with poor prognosis.

**Methods:**

There is a lack of consensus in terms of classification and management algorithm, although lesions greater than 25% of the humeral head had been suggested to need more than a simple Bankart repair to avoid recurrence. The concept of glenoid track has turned the attention to location and shape and not only size of the humeral defect. Moreover, the glenoid bone loss is crucial when choosing a treatment option as it contributes to decrease the glenoid track as well. A thorough revision of treatment options has been performed.

**Results:**

Numerous treatment options have been proposed including remplissage, glenoid or humeral head augmentation, bone desimpaction, humeral rotational osteotomy and arthroplasty.

**Conclusion:**

Humeral defects treatment should be individualized. Determination of size and location of the defect and its relation with glenoid track is mandatory to achieve satisfactory results.

## 1 INTRODUCTION

Malgaigne first described a bony defect of the posterolateral humeral head in 1855 [1]. Later on, in 1940, Hill and Sachs wrote a review article on humeral bony defects after traumatic glenohumeral dislocations, what has given their names to the well-known lesion [2]. It was described as a “line of condensation on the shoulder radiograph when performed in internal rotation” and explained as a compression fracture of the posterior-superior aspect of the humeral head due to an impact against the glenoid rim. Since then, a thorough study of shoulder instability pathophysiology and a better understanding of biomechanics have demonstrated the relevance of this lesion when choosing a treatment option. A correct identification of this injury leads to a well-established decision-making process depending on its size and location and has shown to have a predictive value in recurrence rate.

### 1.1 Epidemiology

The real incidence of Hill Sachs lesion remains unknown. Hill and Sachs reported in their review an incidence of 20% to 100% [2]. Nowadays, the incidence ranges from 32% to 88% of all anterior shoulder instability events and has been associated to high rates of recurrent instability, where it is present in 77% to 100% of the cases [3-6]. It is also well known that dislocations rather than subluxations cause larger Hill-Sachs lesions, as well as repetitive events [7, 8]. When defining the incidence by direct visualization in arthroscopy, the presence of the “bare area” should be taken into account in order to avoid over-diagnosing. It is the area that lies between the insertion of the posterior capsule and the edge of the articular surface, located along the anatomical neck of the humerus and should not be mistaken for a Hill Sachs lesion. Saito *et al.* reported that the most inferior portion of the Hill-Sachs lesion could overlap the bare area when it extended more than 19mm from the top of the humeral head [9].

### 1.2 Pathophysiology

**Fig. (1):**
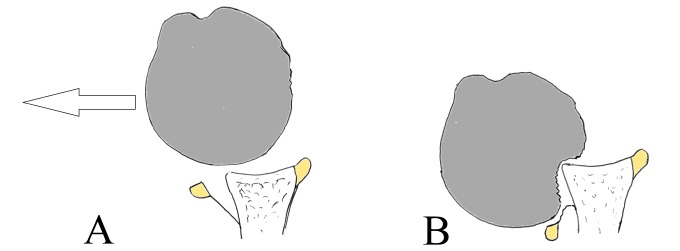
**(A)**Development of the Hill-Sachs defect: As the humeral heads displaces anteriorly during dislocation **(B)**, the anterior glenoid rim will impact with the soft humeral bone, causing the lesion.

Anterior shoulder dislocation typically occurs with the arm in external rotation and abduction. As the humeral head is forced anteriorly, the capsulolabral structures of the shoulder are stretched and often torn [10]. Then, the head subsequently translates farther anteriorly and inferiorly. In this stage, the postero-superior-lateral aspect of the humeral head impacts against the anterior glenoid rim resulting in a compression fracture [2]. As mentioned before, in order to allow the humeral head to dislocate, an anterior detachment of the capsulolabral tissues is mandatory, what is called a Bankart lesion [11]. In other cases, however, equivalent lesions can be present, as for example, glenoid bony injury (Bony Bankart) or periosteal lesions (anterior labro-ligamentous periosteal sleeve avulsion lesion, ALPSA) [4, 7], that can also contribute to recurrent instability patterns. When any of these happen, in each episode, the soft tissues attenuate progressively, allowing more contact between the soft humeral cancellous bone and the hard glenoid rim cortical bone (Fig. **1**). Both mechanisms result in a greater bony defect and a weaker anterior soft tissue restraint, and eventually a higher risk of re-dislocation [12].

**Fig. (2):**
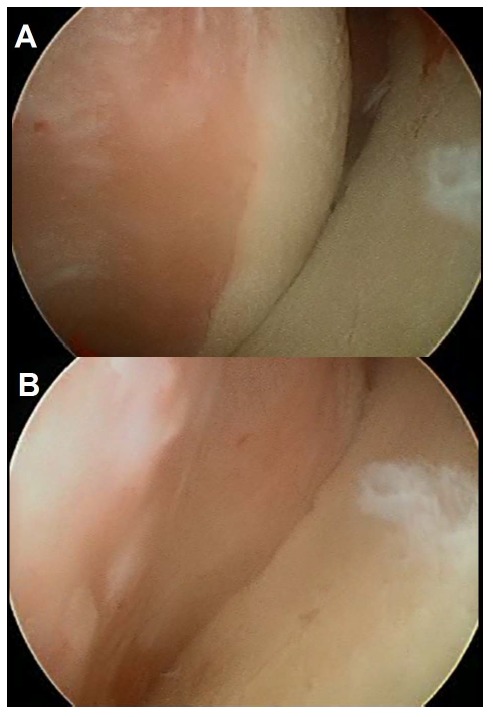
Arthroscopic view of an large engaging Hill- Sachs lesion **(A)** viewed form the posterior portal in a left shoulder. When the humerus is rotated externally the defect slides over the glenoid, “engaging” and causing the dislocation **(B)**.

In 2000, Burkhart and De Beer [13] demonstrated that it is not only size that matters, but also location and shape. Sixty years after the first description of a Hill Sachs lesion, a new concept emerged, called the “engaging Hill Sachs”. It was defined arthroscopically as a humeral head defect that is oriented parallel to the anterior rim and thus engages with it when the shoulder is positioned in the dislocation position. The prevalence of this engaging Hill-Sachs lesion was reported to be 1.5% by Burkhart and De Beer [13], 27% by Pagnani [14], and 34% by Cho *et al.* [15]. A few years later, in 2007, Yamamoto *et al.* introduced the second key concept in order to understand recurrent dislocations, the “glenoid track” [16]. It was described as the contact zone between the glenoid and the humeral head when the arm is elevated in maximum external rotation and maximum horizontal extension and can be expressed in quantitative or qualitative terms. If the Hill-Sachs lesion extends more medially over the medial margin of the glenoid track, there is a risk of engagement and dislocation (Fig. **2**). According to the measurement of Yamamoto *et al.* using cadaveric shoulders, the medial margin of the glenoid track was located at a distance equivalent to 84% of the width of the glenoid. Omori *et al.* also confirmed this measurement with MRI [17]. These authors also demonstrated that the glenoid track was smaller as the degree of abduction increased, reaching a minimum of 81% of the width of the glenoid when the arm was abducted 150º. In summary, as recently demonstrated by Kurokawa *et al.* [18] in a CT study, two different types of engaging Hill Sachs can be identified. The first engaging Hill Sachs injury would be a large and wide bone defect and the second type, a narrow but medially located lesion. These authors described an overall incidence of 7% of engaging Hill-Sachs lesions. Interestingly, in all of the cases, a large glenoid bony defect was present. It could be said that when an osseous glenoid lesion increases in size, the glenoid track decreases accordingly, raising the probability of engaging.

### 1.3 Imaging

In order to evaluate anterior glenohumeral instability, the following three radiological projections should be obtained: an antero-posterior view of the shoulder in internal and external rotation and an axillary view. As mentioned before, the Hill Sachs defect occurs in the posterolateral aspect of the humeral head and can be better demonstrated by a combination of the internal rotation AP view and the Stryker notch view. On the other hand, Didiee and West Point views allow are especially useful when looking for bony Bankart defects [19].

Although MRI continues to be the most used imaging technique (as it is routinely performed in this context to evaluate soft tissue damage), computerized tomography (CT) is widely considered the gold standard for evaluating bone loss Fig. (**3**). Three-dimensional CT reconstructions allow for a precise evaluation of the size (width and depth on axial and coronal images), orientation (Hill-Sachs angle) and location (bicipital and vertical angles) [15]; however, it is not useful for detecting purely cartilaginous lesions [8].

Magnetic resonance (MR) imaging and MR arthrography are the optimal techniques to define soft tissue lesions of the capsule-labral anterior complex. Some authors advocate the use of T1-weighted sequences with fat suppression in axial, oblique sagittal and coronal directions, as well as the ABER (Abduction-External rotation) series [20]. This type of images that are obtained in the prone position for shoulder dislocation, are typically useful in order to detect articular supraspinatus tears and a better identification of the anterior portion of the glenoid labrum and the anterior band of the inferior glenohumeral band [21].

### 1.4 Classification and Clinical Relevance

Several classification systems have been proposed, however, none of them has been yet accepted as a useful management tool. For example, Rowe *et al.* [22] classified the lesion depending on the size as measured in the axillary projection. The Hill Sachs lesion was defined as mild (2cm long x 0.3 cm deep), moderate (2-4 cm long x 0.3-1 cm deep) and severe (>4 cm x > 1cm deep). Calandra *et al.* [3] and Franceschi *et al.* [23] based their classification in severity of the injury on direct visualization, whether or not it affected subchondral bone. More recent studies have focused on the volume of humeral head involved or the percentage of the surface of the articular part of the humeral head, either on direct visualization during arthroscopy or measured in MRI or CT images.

Given the lack of consensus when classifying Hill Sachs lesions together with the fact that soft tissue damage, demographic features and quality of repair can influence the clinical result of the surgery, the question is now whether or not the injury is of clinical relevance. This idea was first proposed by Flatow and Warner [24] and was complemented by Di Giacomo *et al.* recognizing the importance of bipolar bony lesions when applying the glenoid track concept [25].

The average size of the Hill Sachs lesion is 22 mm in width and 5 mm in depth [9]. Saito *et al.* also studied the location on axial CT images, using a clock face (12 o’clock = anterior, 3 o’clock = medial, 6 o’clock = posterior, 9 o’clock = lateral) and described the top of the lesion located more posteriorly (6:46 hours) and the bottom more laterally (average 8:56) [9]. The critical size of the Hill Sachs lesion that can cause instability is a medium or large defect, greater than 20% of the humeral head articular surface, with a depth greater than 16% of the humeral head diameter, a volume greater than 250mm3 or 1000mm3 or those that affect more than 5/8 of the humeral head radius [26]. On the other hand, Sekiya *et al.* in a cadaveric study, demonstrated that although a defect of 25% of the humeral head causes an increase in anterior translation and bony contact force, it was not enough in isolation to cause recurrent anterior instability [27].

### 1.5 Management

A correct assessment and treatment of the bony lesions associated to soft tissue damage in anterior shoulder instability is crucial. The recurrence rate after arthroscopic labral repair in the absence of glenoid or humeral head loss is only 4% [28] but it rises to 67% when substantial glenoid or humeral bone loss is present [13]. It has also been reported that the presence of a Hill-Sachs that could be noticed in a simple Rx with the shoulder in external rotation multiplied the recurrence rate by three (from 10% to 31%) [13].

Traditionally, the different treatment options considered when dealing with a large Hill-Sachs defect were focused in addressing the humeral defect itself (by filling it with bone or tendon or tendon or putting it away with an osteotomy); However, the concept of glenoid track changed the focus to an association of both humeral and glenoid defects. Some authors have advocated that a procedure more focused in the glenoid side that increases the glenoid track, such as a coracoid transfer to the anterior glenoid would be able to reduce the risk of an engaging Hill-Sachs [29]. Nevertheless, the most accepted absolute indications for an specific surgical management of Hill Sachs lesions are: Displaced humeral head fracture-dislocation and an associated Hill Sachs injury or lesions that affect more than 30%-40% of the humeral head with chronic dislocation or recurrent anterior instability. Relative indications would include: lesions of 20% to 35% of the humeral head with glenoid engagement on examination, lesions of more than 20% of the articular surface and signs of humeral head engagement on examination and lesions of 10% to 25% of the humeral head in the cases when the humeral head does not remain well centered in the glenoid fossa after arthroscopic instability repair [12].

#### 1.6 Conservative Management

A non-surgical approach might be considered in the cases of small osseous defects and non-engaging Hill-Sachs lesions. An appropriate treatment of Bankart lesion and other relevant pathology should be carefully addressed. In these cases, the standard rehabilitation protocol is performed afterwards, with an especial focus in deltoid muscle, rotator cuff muscles and scapular stabilizers strengthening [12].

#### 1.7 Remplissage

**Fig. (4):**
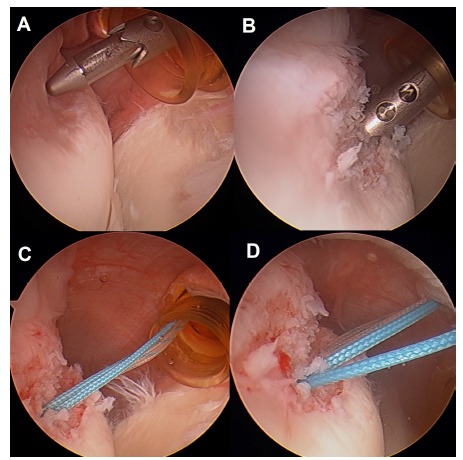
The Remplissage procedure: the lesion is identified from the anterosuperior portal (A). After debridement of the surface (B), two implants are placed along the defect (C) and the corresponding sutures passed percutaneously through the posterior capsule and infraspinatus tendon (D) allowing for an effective capsulotenodesis.

This technique consists in transforming an intra-articular humeral defect in an extra-articular lesion, thus reducing the risk of engaging (Fig. **4**). Remplissage is the French word for “fill-in” and it is used in this context as the technique consists in a posterior capsulodesis and infraspinatus tenodesis into the bony defect area. It was first described by Purchase *et al.* [30] in 2008 and posteriorly modified by Koo *et al.* [31] in 2009 and is indicated in moderate to great humeral head bony defects without glenoid bone loss or combined with a non significant glenoid bone loss.

The arthroscopic technique starts with a posterior vision in order to identify the Hill Sachs lesion, once the standard general evaluation of the joint has been performed [30]. An accessory portal is made just lateral to the posterior portal aiming the area of the lesion.

 After carefully debriding the Hill Sachs zone an implant with two strands is introduced in the cancellous bone, and the two strands are passed independently through the infraspinatus muscle. It can also be performed with two or more implants to create a greater footprint. More recently, the use of all-suture implants has also been advocated. Care should be taken not to place the anchors too medial as it can result in restricted motion [32]. Knots are usually tied in the subacromial space after the anterior capsulolabral repair.

The postoperative management is not different from a simple anterior capsulolabral repair and will consist in 3 weeks of sling followed by passive and active exercises limiting external rotation to 0º until the sixth week. Several authors have demonstrated healing of the capsulotenodesis by means of imaging techniques [33, 34].

Remplissage is an effective procedure and has demonstrated that reduces the rate of re-dislocation from >25% to approximately 5% when a Hill Sachs of 25% of the humeral head is present [29, 34-36]. Wolf *et al.* reported excellent results in their series of 45 patients with medium size humeral defects even in the presence of small glenoid defects. The mean follow up was 4 years and the reported re-dislocation rate of only 4.5% with a minimal rate of complications [37]. Park *et al.* in a longer follow up series found a recurrence rate of 15% after 5 years [38]. However, most of these studies lack of a control group. Ruiz Ibán *et al.* in a paired cohort study compared a group of similar patients with large Hill-Sachs defects that underwent simple Bankart repair with a group of patients in which both procedures were performed. They also reported a decrease in dislocation rate of 17% without finding any differences in range of motion or functional outcome [39].

With regards to clinical outcome, Boileau *et al.* in their series reported a rate to return to sports of 90%, achieving the same level in 68% of the cases, including overhead activities. The rate of stable shoulders after 2 years of follow up was 98% [33]. Park *et al.* found lower WOSI scores at final follow up in the cases when a concomitant lesion (ALPSA; Kim lesion) was present and subsequently addressed during surgery [38].

The complication rate of this procedure is very low (0.9%). Persistent tenosynovitis of the long head of biceps and ipsilateral ulnar nerve palsy have been described [36]. Despite the safety of this technique, it has been related to stiffness of the joint and limited range of motion due to its non-anatomic nature. Boileau *et al.* reported a loss of external rotation of approximately 10º in their series [33]. However, when comparing final range of movement after undergoing simple Bankart repair and Bankart repair with an associated remplissage technique, a similar loss of external rotation can be expected in both groups [39]. This finding could suggest that the loss of external rotation might not be attributed only to this technique. Moreover, Merolla *et al.* have reported that infraspinatus muscle strength is recovered satisfactory when comparing to healthy subjects [40].

## 2 GLENOID BONE AUGMENTATION

**Fig. (5):**
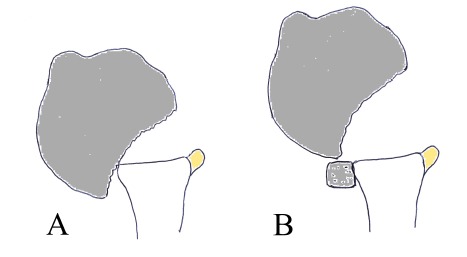
Biomechanical rationale for anterior glenoid bone augmentation: in a patient with a significant humeral bony defect **(A)** supplementation of the anterior glenoid rim will avoid engaging of the Hill-Sachs lesion **(B)**.

Glenoid bone augmentation is a well-documented technique for addressing recurrent instability in the presence of an anterior glenoid defect [13, 41]. The most common procedures include coracoid transfer and iliac bone grafting. However, as previously mentioned some authors also advocate the use of these procedures in the presence of a Hill Sachs lesion in order to increase the glenoid track and reduce the risk of engagement (Fig. **5**) [29, 42, 43].

## 3 HUMERAL HEAD BONE AUGMENTATION

The aim of this technique is to restore the anatomy of the humeral head by filling the defect created by the impaction of the glenoid [44]. This would eventually avoid engaging of the lesion. The main indications are a humeral defect of more than 40% of the humeral head in the absence of glenoid bone loss or a failed Bankart repair in the presence of a humeral defect greater than 25% [36, 45]. Bone plugs are custom-made for each defect using an autograft, an allograft Fig. (**6**) or a synthetic material [45-48]. In exceptional occasions a fresh humeral osteoarticular graft can be used to replace the entire humeral head. The potential advantages of this procedure are the anatomical nature of the reconstruction, the restoration of range of movement and the avoidance of a replacement in extreme cases. The downsides are the necessity of an open approach, the risks of osteonecrosis of the humeral, graft resorption, nonunion or hardware complications [46]. Only case series and case reports have been published [49]. The complication rate is as high as 20% to 30% with a reoperation rate of more than 25% of the patients.

## 4 DESIMPACTION

Desimpaction of the humeral head is relative new techniques that consist in elevating the compressed bone and support it with bone graft in an attempt to restore native anatomy [50-52]. The main indication would be an acute lesion of less than 3 weeks of evolution and less than 40% of involvement of the articular surface [12]. It can be performed using a cortical window in the mid greater tuberosity just lateral to the bicipital groove and introducing a curved bone tamps in a retrograde fashion until reaching the defect. The correction should be performed and evaluated under fluoroscopic guidance [50]. Alternatively, Re *et al.* described a percutaneous technique helped by an anterior cruciate ligament tibial guide [51]. More recently, the use of an inflatable balloon has also been described [52]. However, only cadaveric studies have been reported and consequently more clinical studies should be performed before recommending these techniques.

## 5 HUMERAL OSTEOTOMY

Humeral osteotomy was first described by Weber and consists in a rotational osteotomy of the proximal humeral shaft in order to improve retroversion of the proximal humerus [53]. It involves a subcapital humeral osteotomy with a medial rotation of the humeral head and imbrication of the subscapularis and anterior capsule [54]. The main retroversion aimed is 20º to 35º and the use of a guide intraoperatively is recommended to confirm the amount of derotation before plating, as it is a technically highly demanding procedure [55, 56]. Kronberg *et al.* studied the influence of diminished humeral retroversion in recurrence rate after Bankart repair [56].

The reported recurrence rate is of 5% to 10%, however, the overall rate of complications and reoperation is high [55]. Complications would include pain (complex regional syndrome), hematoma, infection, delayed malunion, rotation deficit and necessity of hardware removal [36, 53, 56]. Weber *et al.* [53] reported the greatest series of rotational osteotomies, consisting of 180 cases. The recurrence rate that they found was 6.8% without detecting a significant loss of external or internal rotation. Excluding hardware removal, only 9 patients required re-operation for a major complication. However, more recent series have been unable to reproduce their results. Brooks-Hill *et al.* in their series of 25 patients reported that the mean ASES score was 78 points, but 10 of them were unable to wash their backs with the affected arm. Moreover, nine of the 25 patients required a re-intervention [55].

## 6 RESURFACING AND HEMIARTHROPLASTY

Humeral arthroplasty could be indicated in patients with humeral defects greater than 45% of the humeral head. In elderly patients, the existence of osteoporotic bone leads to greater bony defects. Moreover, it is frequent to find a rotator cuff disease and degenerative changes what can be an indication for a hemiarthroplasty, total arthroplasty or even reverse arthroplasty.

 In the clinical scenario of a young patient with a massive Hill Sachs and early degenerative changes, there are not satisfactory options. More conservative options would include partial and complete resurfacing [54]. When comparing partial resurfacing with graft implantation, it is not clear whether it is easier to match the defect with the implant or with the bone plug, as the geometry might be difficult to reproduce [57]. With regards to complete resurfacing and arthroplasty, it might be indicated in the setting of chronic dislocations with Hill Sachs defects greater than 40% [36, 54].

## CONCLUSION

In the context of anterior shoulder instability, humeral defects (Hill-Sachs lesions) have a clear impact in the recurrence rate of the dislocated shoulder and in the success of its surgical management.

It is essential to assess the humeral defects appropriately; this can be done with simple radiographic evaluation or MRI, but CT with 3D reconstruction is considered the best diagnostic procedure. During evaluation, attention should be paid not only to the width, length and depth of the defect but also to its position and orientation, trying to define if the defect can easily converge with the anterior rim of the glenoid during abduction and external rotation (an engaging Hill-Sachs). This can happen even with smaller defects when an associated glenoid defect is present, thus the glenoid track has to be carefully assessed.

Larger lesions and engaging lesions should be addressed specifically during surgery. Many alternatives have been proposed such as allo or autograft reconstruction, desimpaction of the compressed bone, humeral osteotomies and even arthroplasty but there are only significant reports of efficacy with the remplissage technique. This procedure, a posterior capsule-tenodesis of the infraspinatus over the defect has shown to lower the recurrence rate of patients with larger defects to levels similar to patients without relevant defects and has a low complication rate.

## Figures and Tables

**Fig. (3):**
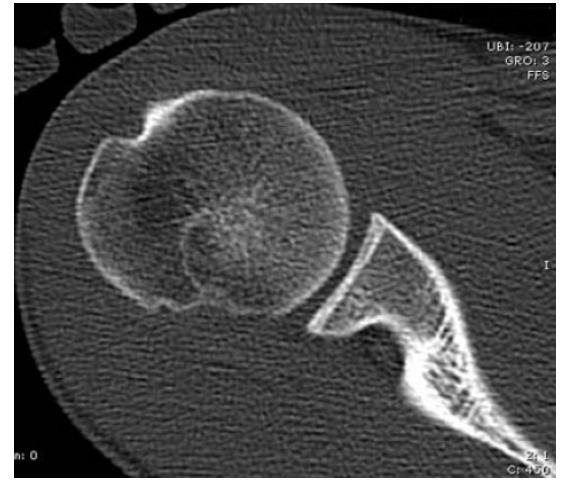
Computed tomography of the shoulder of a patient with shoulder instability showing a small Hill-Sachs lesion.

**Fig. (6):**
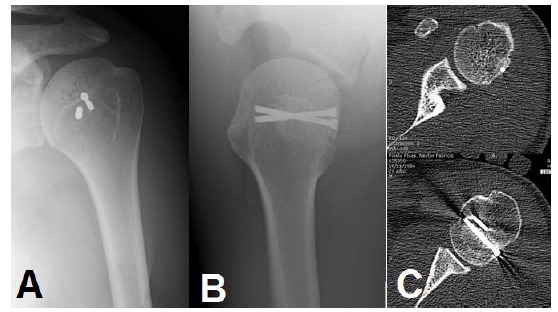
Humeral head augmentation: An osteochondral allograft is placed in the humeral defect and fixed with 2 headless compression screws **(A,B)**. The CT scan shows adequate integration of the defect and restoration of the concavity **(C)**.

## LIST OF ABBREVIATIONS

ABER: = Abduction-external rotation
ALPSA: = Anterior labro-ligamentous periosteal sleeve avulsion lesion
ASES: = American Shoulder and Elbow Score
CT: = Computerized tomography
WOSI: = Western-Ontario Shoulder Instability Score
